# Factors affecting the live-birth rate in women with diminished ovarian reserve undergoing IVF-ET

**DOI:** 10.1007/s00404-018-4884-4

**Published:** 2018-09-19

**Authors:** Yun Huang, Jingyi Li, Fang Zhang, Yifeng Liu, Gufeng Xu, Jing Guo, Runjv Zhang, Yiqing Wu, Juan Liu, Kai Chen, Wei Zhao, Wei Wu, Yanjun Hu, Guangdi Chen, Dan Zhang

**Affiliations:** 1 Key Laboratory of Reproductive Genetics (Ministry of Education), and Department of Reproductive Endocrinology, Women’s Hospital, Zhejiang University School of Medicine, Hangzhou, 310006 People’s Republic of China; 2 Department of Public Health, Institute of Environmental Health, Zhejiang University School of Medicine, Hangzhou, People’s Republic of China

**Keywords:** Diminished ovarian reserve, Live-birth rate, In vitro fertilization and embryo transfer, Impact factors, Cohort study

## Abstract

**Purpose:**

The occurrence of diminished ovarian reserve (DOR) in women was growing in recent years. Although in vitro fertilization and embryo transfer (IVF-ET) became an effective treatment for DOR, the live-birth (LB) rate remains unsatisfactory. This study aimed to investigate the impact factors of LB rate in women with DOR undergoing assisted reproduction.

**Methods:**

This was a single-center retrospective cohort study. A total of 2277 IVF-ET or ICSI cycles from 1957 DOR women were analysed. Impact factors of LB rate were explored via Student’s *t* test, Pearson’s Chi-square test, and multivariate logistic regression models.

**Results:**

There were statistically significant differences in maternal age (*P *< 0.001), duration of infertility (*P *< 0.001), female body mass index (*P *= 0.039), first IVF cycle (*P *= 0.004), poor ovarian response (*P *< 0.001), paternal age (*P *< 0.001), total gonadotropin dose (*P *= 0.010), endometrial thickness (*P *= 0.021), number of follicles ≥ 14 mm (*P *= 0.007), number of oocytes retrieved (*P *< 0.001), number of frozen embryos (*P *= 0.014), and the stage (*P *< 0.001) and number (*P *< 0.001) of embryos transferred between the non-live-birth (NLB) and LB groups. However, only factors of maternal age, the stage and number of embryos transferred remained different after adjusting for potential confounders.

**Conclusions:**

Maternal age, the stage and number of embryos transferred were independent impact factors affecting the live-birth rate in women with DOR seeking for assisted conception.

## Introduction

Ovarian reserve plays a crucial role in menstrual regularity and achieving pregnancy in reproductive-age women. A large number of single or various combined tests have been used for evaluation of the ovarian reserve in an effort to predict the reproductive competence (ovarian response and pregnancy outcome) [1]. The ideal ovarian reserve tests (ORT) could accurately reflect oocytes’ reproductive capacity, and is useful in assisting infertile women with reproductive life planning. Among these ORTs, antral follicle count (AFC), basal serum follicle stimulating hormone (FSH) level, anti-Mullerian hormone (AMH) together with maternal age seem to be the most commonly used tests for detection of ovarian reserve [2]. Diminished ovarian reserve (DOR), with an imprecise definition of a decreased number or quality of oocytes, affects approximately 10% of women seeking fertility treatment [3]. It is associated with poor ovarian response (POR) to controlled ovarian hyperstimulation, and is much more prevalent than premature ovarian failure (POF). The most popular tests used for diagnosing DOR nowadays are AFC and FSH [4]. Other risk or protective factors like maternal age, inhibin B, AMH, ovarian volume, a number of dynamic tests also make contribution to detect DOR [5].

However, few has been known about its etiology until now. It has been reported that there was an association between ABO blood type and DOR occurrence [6, 7]. A retrospective analysis involving 35,479 women who underwent in vitro fertilization and embryo transfer (IVF-ET) cycles showed that women with B antigen (blood type AB or B) were more likely to have DOR [8]. Weghofer et al. reported that there was an association between the age of menarche and DOR risk later in life among infertile women [9]. A recent retrospective case–control study which evaluated differences among causes of DOR showed that DOR women by surgery for endometrioma(s) had significantly lower pregnancy and live-birth rates per cycle compared with patients with idiopathic DOR [10].

Despite of being investigated widely, precise definition and optimal management of DOR remain an unsolved enigma, with no single beneficial intervention having been proposed [11]. Although DOR affects a large portion of the infertile population, there were few studies focusing on the factors influencing their LB rates with assisted reproductive technology (ART).

In the present study, we conducted a retrospective cohort study to investigate the impact factors of live-birth rate in Chinese women with DOR undergoing assistant reproduction, which might shed light on the clinical strategy for DOR women to improve their take-home baby rates.

## Materials and methods

### Study population

Our study is a retrospective, university hospital-based cohort study, carried out at Women’s Hospital, Zhejiang University School of Medicine. Referring to the Bologna Criteria published in 2011 and clinical experience [12], the inclusion criteria for women with defined diminished ovarian reserve (DOR) in this study were women undergoing fresh IVF or intracytoplastic sperm injection (ICSI) with (1) age ≥ 40 years on day 1 of gonadotropin stimulation, and/or (2) serum basal follicle stimulating hormone (bFSH) level ≥ 10 IU/L, and/or (3) bilateral antral follicle count (AFC) < 5 follicles.

To minimize the potential confounding factors, exclusion criteria of subjects were as follows: (1) donor oocytes/sperm cycles, or (2) embryo recipient cycles, or (3) women who applied for pre-implantation genetic diagnosis/screening (PGD/PGS), or (4) women with unilateral ovary, chronic hypertension, diabetes, or heart disease, or (5) women with canceled or all-frozen embryo transfer cycles.

A total of 2277 cycles from 1957 DOR women who underwent IVF-ET or ICSI cycles between January 2010 and December 2014 were enrolled from our medical database. Available information on the dataset included maternal factors (maternal age, age of menarche, body mass index, duration of infertility, first IVF cycle,poor ovarian response, antral follicle count, serum hormone levels, and type of infertility), paternal factors (paternal age, semen volume, and sperm motility), ART procedure (type of ART, total gonadotropin dose, duration of ovarian stimulation, endometrial thickness, number of follicles ≥ 14 mm, number of oocytes retrieved, number of frozen embryos, and the number and stage of embryos transferred), and ART outcomes (clinical pregnancy, live birth, pre-term birth, multiple pregnancies, low birth weight, and miscarriages).

The strategy of our reproductive center on the number of embryos transferred is in accordance with the regulation by Chinese government. Usually, no more than two embryos were transferred. And elective single embryo transfer is suggested when the woman is young (< 30 years), having more than three good-quality embryos, and having no unfavorable prognosis factors, e.g., recurrent implantation failure or recurrent miscarriage. However, three embryos were sometimes allowed to be transferred in women older than 35 years with prior unsuccessful transfer cycle, after counseling the patient on the risk of multiple gestation.

All the clinical and laboratory data were extracted from the historical medical records based on the Hospital Information System (HIS) in Women’s Hospital, Zhejiang University School of Medicine. All private information of names, residential addresses, telephone numbers and Resident Identification Card numbers were kept strictly confidential and were masked in the data analysis. Specific subject could not be identified. The Research Ethics Board of Women’s Hospital, Zhejiang University School of Medicine granted ethics approval and waived informed consent.

### Outcome measures

Primary outcome was live-birth rate. Secondary measures consisted of clinical pregnancy (CP), pre-term birth (PB), multiple pregnancies (MP), miscarriages (M), and low birth-weight (LBW) rates. Serum β human chorionic gonadotropin (β-hCG) was tested 14 days after embryo transfer, and for those with a positive β-hCG test, ultrasonography was performed 35 days after embryo transfer.

We defined the pregnancy outcomes in this study according to “International Committee for Monitoring Assisted Reproductive Technology (ICMART) and the World Health Organization (WHO) Revised Glossary of ART Terminology, 2009” [13]:

1. Clinical pregnancy: serological β-hCG positive 14 days after embryo transfer, and after transplantation ultrasonography visualization of one or more gestational sacs, including ectopic pregnancy, after 30–35 days of transplantation.
2. Implantation rate: the ratio of the number of embryos implanted to the total number of embryos transferred.
3. Clinical pregnancy rate: the ratio of the number of clinical pregnancy cycles to the total number of transfer cycles.
4. Miscarriage: identified as clinical pregnancy, but the pregnancy spontaneously terminated in less than 28 weeks. Early miscarriage occurred in less than 14 weeks.
5. Miscarriage rate: the ratio of the number of miscarriage cycles to the total number of transfer cycles.
6. Preterm delivery: delivered after 28 weeks but before 37 weeks.
7. Low birth weight (LBW): birth weight less than 2500 g.

### Statistical analysis

Statistical analyses were performed with Statistical Package for the Social Sciences software (SPSS version 20.0; IBM/SPSS, Inc.). The continuous variables were summarized as mean ± standard deviation (SD) and were tested by unpaired Student’s *t* test. Dichotomous data were shown as percentages. Differences between categorical variables were tested using Pearson’s Chi-square test. The characteristics that were significantly different (*P *< 0.05) between the LB and non-live-birth (NLB) groups were then selected to construct logistic regression models. Both univariable and multivariate logistic regression models were employed to evaluate the impact of variables of interest on odds of live birth after assisted reproduction in women with DOR. Two-sided *P* values of less than 0.05 were considered statistically significant.

## Results

**Fig. 1:**
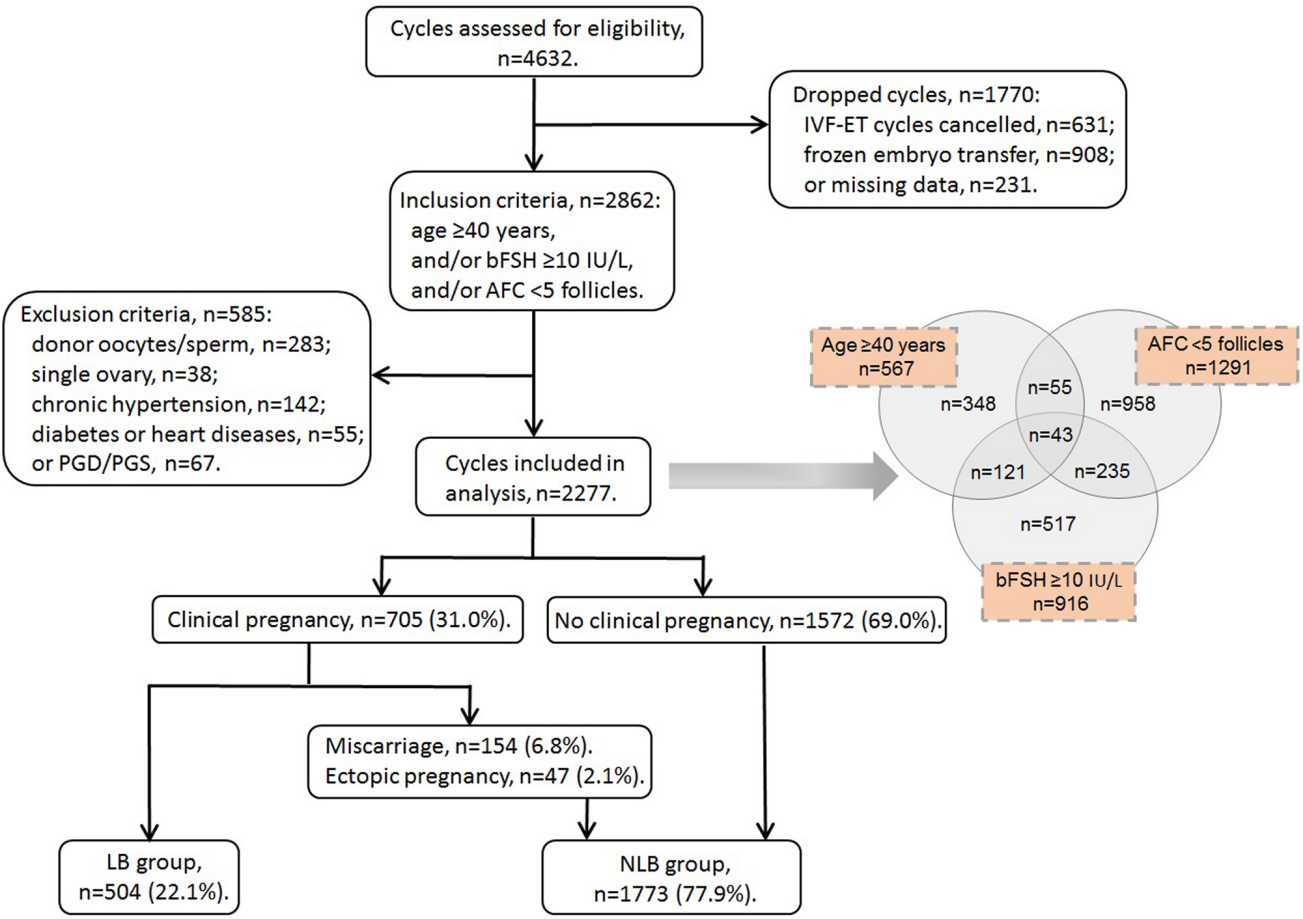
Flow chart of context diagram in the study. *IVF-ET* in vitro fertilization-embryo transfer, *PGD* pre-implantation genetic diagnosis, *PGS* pre-implantation genetic screening, *bFSH* basal follicle stimulating hormone, *AFC* antral follicle count

Totally, 4632 cycles were assessed for eligibility during January 2010–December 2014 (Fig. 1). We dropped those with IVF-ET cycles canceled (*n* = 631), frozen embryo(s) transferred (*n* = 908) and missing data on follow-up (*n* = 231), as well as those meeting the exclusion criteria of donor oocytes/sperm (*n* = 283), single ovary (*n* = 38), chronic hypertension (*n* = 142), diabetes mellitus or heart diseases (*n* = 55) and PGD/PGS (*n* = 67). Finally, 2277 cycles were remained for analyses, and were divided into two groups, including live-birth group (LB, *n* = 504) and non-live-birth group (NLB, *n* = 1773). All the eligible cycles consisted of 567 women aged at least 40 years, 1291 women with bilateral AFC counts of < 5 follicles and 916 women with bFSH levels of ≥ 10 IU/L.

### Baseline characteristics and clinical outcomes of DOR women undergoing IVF or ICSI grouped according to live birth

**Fig. 2:**
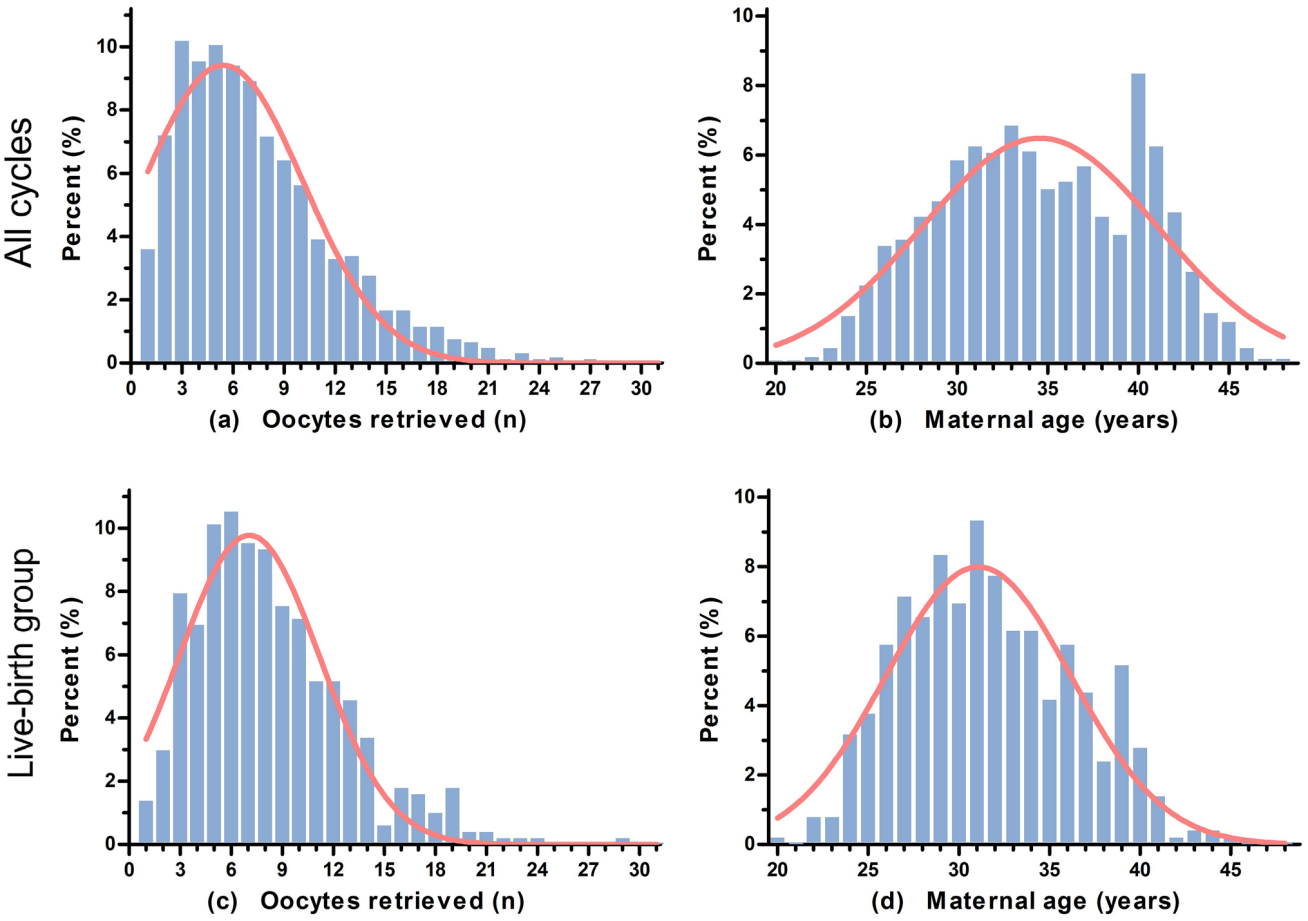
Distributions of oocytes retrieved and maternal age in all studied cycles and the live-birth (LB) group. **a** Distribution of oocytes retrieved in all studied cycles; **b** distribution of maternal age in all studied cycles; **c** distribution of oocytes retrieved in the LB group; **d** distribution of maternal age in the LB group

As shown in Table 1, we found statistically significant differences in maternal age (35.06 ± 5.53 vs 32.61 ± 4.70 years, *P *< 0.001), duration of infertility (5.41 ± 4.19 vs 4.67 ± 3.45 years, *P *< 0.001), female body mass index (BMI, 22.31 ± 2.87 vs 22.01 ± 3.11 kg/m^2^, *P *= 0.039), first IVF cycle (*P *= 0.004), poor ovarian response (*P *< 0.001), paternal age (36.78 ± 6.16 vs 34.55 ± 5.48 years, *P *< 0.001), total gonadotropin dose (2610.74 ± 1097.89 vs 2494.07 ± 819.95 IU, *P *= 0.010), endometrial thickness on the day of human chorionic gonadotropin (hCG) triggering injection (10.38 ± 2.17 vs 10.63 ± 2.25 mm, *P *= 0.021), number of follicles ≥ 14 mm on triggering day (5.80 ± 3.72 vs 6.31 ± 3.20, *P *= 0.007), number of oocytes retrieved (7.24 ± 4.76 vs 8.30 ± 4.42, *P *< 0.001), number of frozen embryos (0.83 ± 1.77 vs 1.12 ± 1.91, *P *= 0.014), and the stage (*P *< 0.001) and number of embryos transferred (*P *< 0.001) between the NLB and LB groups. The median number of oocytes retrieved was 7 [inter-quartile range (IQR) 4–10; Fig. 2a], and the median maternal age was 34 years (IQR 30–39; Fig. 2b) in all studied cycles. Distributions of the number of oocytes retrieved and maternal age in the LB group were elucidated in Fig. 2c, d. Nevertheless, we found no statistically significant differences in the age of menarche, AFC, basal serum levels of follicle stimulating hormone (FSH), luteinizing hormone (LH), estradiol (E_2_), progesterone (P) and testosterone (T), semen volume, sperm motility [represented by the rate of progressive relativity (PR) in sperm], type of ART, type of infertility, duration of ovarian stimulation, transfer embryo(s) at least one 8CII rate, and serum P and E_2_ levels on triggering day between the study groups (*P *> 0.05, respectively).

**Table 1:** Baseline characteristics and IVF outcomes of DOR women undergoing IVF-ET

	All (*n* = 2277)	NLB (*n* = 1773)	LB (*n* = 504)	*P* value
Maternal age (years, mean ± SD)	34.52 ± 5.46	35.06 ± 5.53	32.61 ± 4.70	< 0.001
Age at menarche (years, mean ± SD)	14.44 ± 1.32	14.36 ± 1.32	14.79 ± 1.29	0.096
Body mass index (kg/m^2^, mean ± SD)	22.25 ± 2.93	22.31 ± 2.87	22.01 ± 3.11	0.039
Duration of infertility (years, mean ± SD)	5.24 ± 4.05	5.41 ± 4.19	4.67 ± 3.45	< 0.001
First IVF cycle				0.004
Yes [*n* (%)]	1614 (70.9)	1231 (69.4)	383 (76.0)	
No [*n* (%)]	663 (29.1)	542 (30.6)	121 (24.0)	
Poor ovarian response				< 0.001
Yes [*n* (%)]	478 (21.0)	416 (23.5)	62 (12.3)	
No [*n* (%)]	1799 (79.0)	1357 (76.5)	442 (87.7)	
Antral follicle count (*n*, mean ± SD)	4.98 ± 3.26	5.01 ± 3.32	4.88 ± 3.06	0.438
Basal FSH (IU/L, mean ± SD)	9.16 ± 3.91	9.20 ± 3.81	9.01 ± 4.27	0.336
Basal LH (IU/L, mean ± SD)	4.88 ± 2.63	4.87 ± 2.58	4.93 ± 2.78	0.638
Basal E_2_ (pmol/L, mean ± SD)	136.42 ± 91.79	138.21 ± 90.02	130.14 ± 97.57	0.082
Basal P (nmol/L, mean ± SD)	1.74 ± 1.12	1.72 ± 1.14	1.78 ± 1.03	0.408
Basal T (nmol/L, mean ± SD)	0.86 ± 1.03	0.84 ± 1.00	0.92 ± 1.12	0.218
Paternal age (years, mean ± SD)	36.28 ± 6.08	36.78 ± 6.16	34.55 ± 5.48	< 0.001
Semen volume (ml, mean ± SD)	2.94 ± 1.94	2.98 ± 2.12	2.81 ± 1.12	0.602
The rate of PR in sperm (%)	40.54 ± 13.82	40.25 ± 13.77	41.59 ± 14.03	0.379
Type of ART				0.058
IVF cases [*n* (%)]	1749 (76.8)	1346 (75.9)	403 (80.0)	
ICSI cases [*n* (%)]	528 (23.2)	427 (24.1)	101 (20.0)	
Type of infertility				0.987
Primary infertility [*n* (%)]	808 (35.5)	629 (35.5)	179 (35.5)	
Secondary infertility [*n* (%)]	1469 (64.5)	1144 (64.5)	325 (64.5)	
Total gonadotropin dose (IU, mean ± SD)	2585.06 ± 1044.05	2610.74 ± 1097.89	2494.07 ± 819.95	0.010
Duration of stimulation (days, mean ± SD)	9.45 ± 2.28	9.44 ± 2.35	9.47 ± 1.99	0.750
Endometrial thickness on day of hCG (mm, mean ± SD)	10.43 ± 2.19	10.38 ± 2.17	10.63 ± 2.25	0.021
P levels on triggering day (nmol/L, mean ± SD)	2.22 ± 1.09	2.23 ± 1.11	2.19 ± 1.00	0.387
E_2_ levels on triggering day (pmol/L, mean ± SD)	8477.49 ± 5800.58	8405.62 ± 5956.00	8731.67 ± 5211.58	0.240
Number of follicles ≥ 14 mm (*n*, mean ± SD)	5.91 ± 3.62	5.80 ± 3.72	6.31 ± 3.20	0.007
Number of oocytes retrieved (*n*, mean ± SD)	7.47 ± 4.71	7.24 ± 4.76	8.30 ± 4.42	< 0.001
Embryo stage				< 0.001
Day 2 [*n* (%)]	815 (35.8)	692 (39.0)	123 (24.4)	
Day 3 [*n* (%)]	1462 (64.2)	1081 (61.0)	381 (75.6)	
Number of embryos transferred				< 0.001
One [*n* (%)]	490 (21.5)	443 (25.0)	47 (9.3)	
Two [*n* (%)]	1103 (48.4)	810 (45.7)	293 (58.1)	
Three [*n* (%)]	684 (30.0)	520 (29.3)	164 (32.5)	
Transfer embryo(s) at least one 8CII				0.546
Yes [*n* (%)]	1057 (46.4)	829 (46.8)	228 (45.2)	
No [*n* (%)]	1220 (53.6)	944 (53.2)	276 (54.8)	
Number of frozen embryos (*n*, mean ± SD)	0.90 ± 1.81	0.83 ± 1.77	1.12 ± 1.91	0.014

*SD* standard deviation, *DOR* diminished ovarian reserve, *ART* assisted reproductive technology, *IVF-ET* in vitro fertilization-embryo transfer, *IVF* in vitro fertilization, *ICSI* intracytoplasmic sperm injection, *LB* live birth, *NLB* non-live birth, *FSH* follicle stimulating hormone, *LH* luteinizing hormone, *E*_*2*_ estradiol. *P* progesterone, *T* testosterone, *hCG* human chorionic gonadotropin, *PR* progressive relativity

### Relationship between the impact factors affecting LB in DOR women undergoing IVF or ICSI

In subsequent analyses, we performed univariable and multivariate logistic regression analyses to assess if those impact factors remained different between the two groups after adjusting for potential confounders (Table 2). In this regression analysis, we accounted for thirteen variables that were unevenly distributed (*P *< 0.05) between the LB and NLB groups in Table 1. After adjusting for potential confounders, factors of BMI [adjusted odds ratio (aOR) 1.02, 95% confidence interval (CI) 0.97–1.06; *P *= 0.461], duration of infertility (aOR 0.99, 95% CI 0.95–1.03; *P *= 0.542), paternal age (aOR 0.98, 95% CI 0.95–1.01; *P *= 0.188), total gonadotropin dose (aOR 0.92, 95% CI 0.81–1.04; *P *= 0.167), endometrial thickness on the day of hCG triggering injection (aOR 1.03, 95% CI 0.97–1.09; *P *= 0.322), number of follicles ≥ 14 mm on triggering day (aOR 0.97, 95% CI 0.92–1.03; *P *= 0.382), number of oocytes retrieved (aOR 0.98, 95% CI 0.93–1.04; *P *= 0.540), first IVF cycle (aOR 1.30, 95% CI 0.94–1.79; *P *= 0.109),poor ovarian response (aOR 0.91, 95% CI 0.57–1.44; *P *= 0.686), and the number of frozen embryos (aOR 1.09, 95% CI 1.00–1.18; *P *= 0.060) were not significantly associated with live birth among DOR women undergoing IVF or ICSI. Meanwhile, we found that compared with subjects with age of ≤ 30 years, those in the age group of ≥ 40 years (aOR 0.26, 95% CI 0.14–0.47; *P *< 0.001) suffered from decreased LB rate. Besides, cycles with embryo stage of day 3 were related with increased likelihoods of LB (aOR 1.53, 95% CI 1.13–2.08; *P *= 0.006). When set women with one embryo transferred as reference, the fully aORs (95% CI) of LB were 2.53 (1.62–3.97) and 4.59 (2.73–7.70) for two and three embryos transferred, respectively. Hence, factors of maternal age, the stage and number of embryos transferred may independently affect the IVF-ET live-birth rates in women with DOR.

**Table 2:** Odd ratios for live birth in DOR women undergoing IVF-ET

	Crude OR (95% CI)	*P* value	Adjusted OR (95% CI)	Adjusted *P* value
Maternal age (years)				
≤ 30	Reference		Reference	
31–34	0.80 (0.62–1.03)	0.083	0.87 (0.60–1.25)	0.442
35–39	0.60 (0.46–0.78)	< 0.001	0.74 (0.46–1.18)	0.203
≥ 40	0.23 (0.16–0.32)	< 0.001	0.26 (0.14–0.47)	< 0.001
Body mass index (kg/m^2^)	0.96 (0.93–1.00)	0.039	1.02 (0.97–1.06)	0.461
Duration of infertility (years)	0.95 (0.93–0.98)	< 0.001	0.99 (0.95–1.03)	0.542
Paternal age (years)	0.94 (0.92–0.96)	< 0.001	0.98 (0.95–1.01)	0.188
Total gonadotropin dose (IU)	0.92 (0.84–1.01)	0.064	0.92 (0.81–1.04)	0.167
Endometrial thickness on day of hCG (mm)	1.06 (1.01–1.10)	0.021	1.03 (0.97–1.09)	0.322
Number of follicles ≥ 14 mm (n)	1.04 (1.01–1.07)	0.009	0.97 (0.92–1.03)	0.382
Number of oocytes retrieved (*n*)	1.05 (1.03–1.07)	< 0.001	0.98 (0.93–1.04)	0.540
First IVF cycle (*n*)	1.39 (1.11–1.75)	0.004	1.30 (0.94–1.79)	0.109
Poor ovarian response (*n*)	0.46 (0.34–0.61)	< 0.001	0.91 (0.57–1.44)	0.686
Number of frozen embryos (*n*)	1.08 (1.02–1.15)	0.010	1.09 (1.00–1.18)	0.060
Embryo stage (*n*)				
Day 2	Reference		Reference	
Day 3	1.98 (1.58–2.48)	< 0.001	1.53 (1.13–2.08)	0.006
Number of embryos transferred (*n*)				
One	Reference		Reference	
Two	3.41 (2.45–4.74)	< 0.001	2.53 (1.62–3.97)	< 0.001
Three	2.97 (2.10–4.21)	< 0.001	4.59 (2.73–7.70)	< 0.001

Multivariate logistic regression analysis was conducted accounting for potential confounders within the cycles performed in our center

*OR* odds ratio, *CI* confidence interval, *DOR* diminished ovarian reserve, *IVF-ET* in vitro fertilization and embryo transfer, *hCG* human chorionic gonadotropin

### Comparisons of ART outcomes in DOR women stratified by maternal age, embryo stage, and the number of embryos transferred

**Fig. 3:**
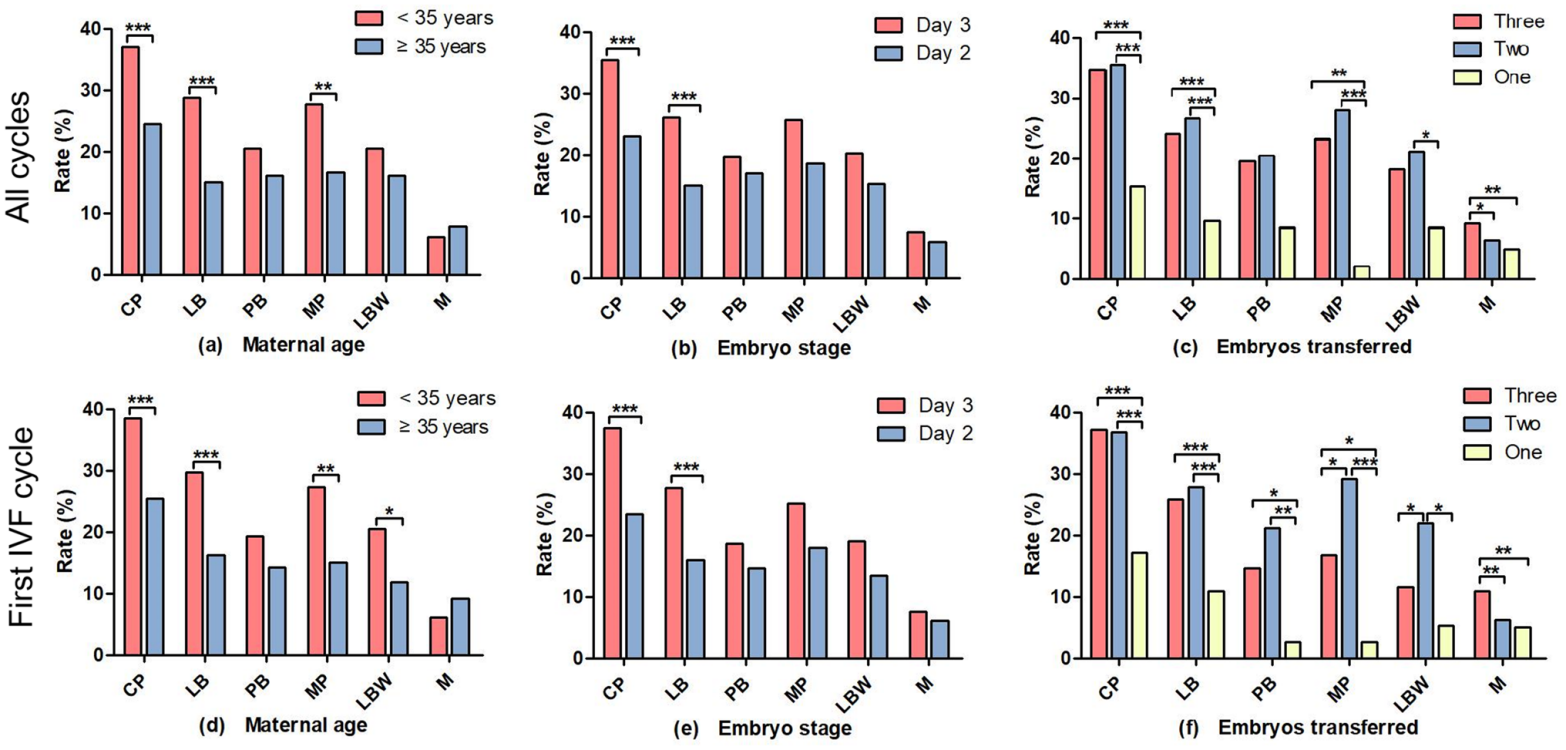
Comparisons of ART outcomes in DOR women stratified by maternal age, embryo stage, and the number of embryos transferred. *CP* clinical pregnancy, *LB* live birth, *PB* pre-term birth, *MP* multiple pregnancies, *LBW* low birth weight, *M* miscarriages, *ART* assisted reproductive technology, *DOR* diminished ovarian reserve. **a**, **d** Comparison of ART outcomes in DOR women stratified by maternal age; **b**, **e** comparison of ART outcomes in DOR women stratified by embryo stage; **c**, **f** comparison of ART outcomes in DOR women stratified by the number of embryos transferred. **a**–**c** In the 2277 cycles; **d**–**f** in the 1614 first IVF cycles. *P < 0.05; **P < 0.01; ***P < 0.001

**Fig. 4:**
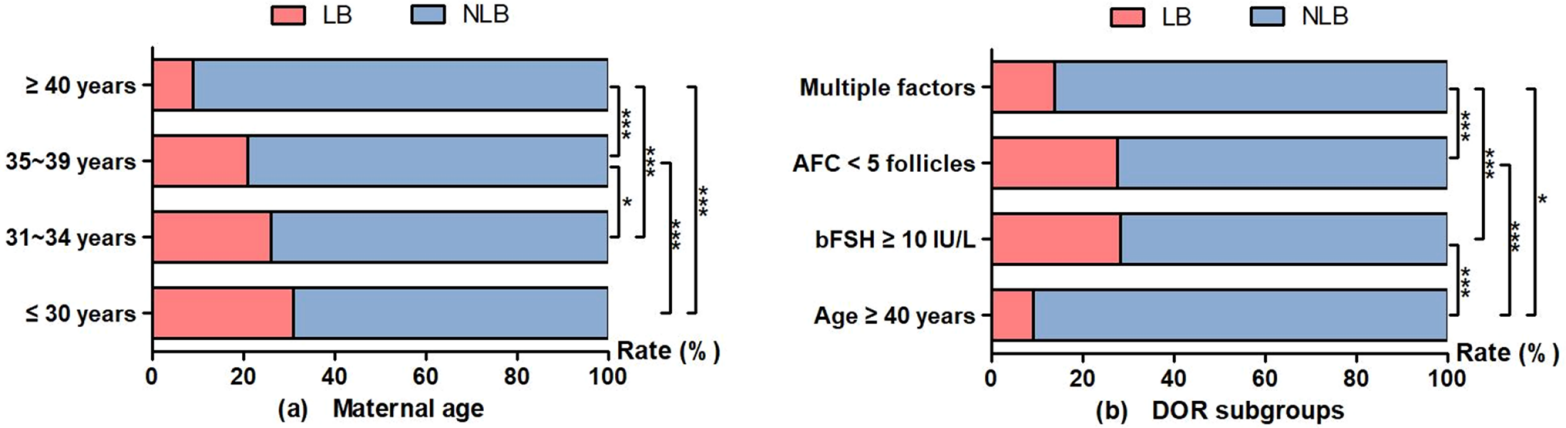
LB rates of DOR women according to different age groups and DOR subgroups. *LB* live birth, *NLB* non-live birth, *DOR* diminished ovarian reserve, *bFSH* basal follicle stimulating hormone, *AFC* antral follicle count. **a** The LB rates were 31.0%, 26.4%, 21.2%, and 9.3% among DOR women in the age groups of ≤ 30 years, 31–34 years, 35–39 years, and ≥ 40 years, respectively. **b** The LB rates were 9.2%, 28.2%, 27.5%, and 13.9% in the DOR subgroups of age ≥ 40 years, bFSH ≥ 10 IU/L, AFC < 5 follicles, and multiple factors, respectively. *P < 0.05; ***P < 0.001

We further conducted stratification analyses by maternal age, and the stage and number of embryos transferred to compare ART outcomes (including CP, clinical pregnancy; LB, live birth; PB, pre-term birth; MP, multiple pregnancies; LBW, low birth weight; M, miscarriages) in DOR women (Fig. 3). Either in the 2277 cycles or in the 1614 first IVF cycles, the CP, LB, and MP rates were statistically higher in the < 35 years age group compared with the ≥ 35 years age group (Fig. 3a, d); the CP and LB rates were statistically higher in the day 3 embryo stage group compared with the day 2 group (Fig. 3b, e). There were significantly higher CP and LB rates among patients transferred with three or two embryos compared to those with one embryo; however, the multiple pregnancies and miscarriages rates also increased (Fig. 3c, f). The LB rates were 31.0% (184/593), 26.4% (152/575), 21.2% (115/542), and 9.3% (53/567) among DOR women in the age groups of ≤ 30 years, 31–34 years, 35–39 years, and ≥ 40 years, respectively (Fig. 4a), and the LB rates were 9.2% (32/348), 28.2% (146/517), 27.5% (263/958), and 13.9% (63/454) in the DOR subgroups of age ≥ 40 years, bFSH ≥ 10 IU/L, AFC < 5 follicles, and multiple factors, respectively (Fig. 4b).

### Comparisons of the number of embryos available for transfer in DOR women according to clinical pregnancy and live birth

**Fig. 5:**
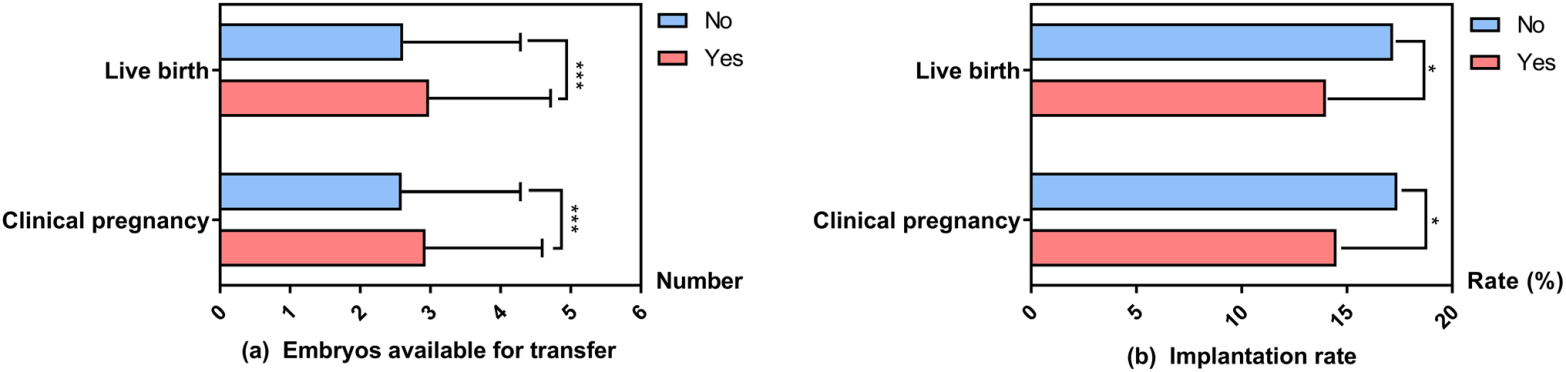
Comparisons of the number of embryos available for transfer and implantation rates in DOR women according to clinical pregnancy and live birth. *DOR* diminished ovarian reserve. **a** There were statistically significant differences in the number of embryos available for transfer between the live birth and non-live birth groups (2.98 ± 1.73 vs 2.61 ± 1.67, *P *< 0.001) and the clinical pregnancy and non-clinical pregnancy groups (2.93 ± 1.66 vs 2.59 ± 1.69, *P *< 0.001). **b** There were statistically significant differences in implantation rates between the live birth and non-live birth groups (14.0% vs 17.2%, *P *= 0.010) and the clinical pregnancy and non-clinical pregnancy groups (14.5% vs 17.4%, *P *= 0.011). *P < 0.05, ***P < 0.001

As the number of surplus embryos showed borderline significance, we further performed analyses on the number of embryos available for transfer (embryos that were transferred plus those that were frozen) according to clinical pregnancy and live birth. As shown in Fig. 5a, there were statistically significant differences in the number of embryos available for transfer between the live birth and non-live birth groups (2.98 ± 1.73 vs 2.61 ± 1.67, *P *< 0.001) and the clinical pregnancy and non-clinical pregnancy groups (2.93 ± 1.66 vs 2.59 ± 1.69, *P *< 0.001). We also conducted an analysis on the implantation rate (number of embryos implanted/total number of embryos transferred). As shown in Fig. 5b, there were statistically significant differences in implantation rates between the live birth and non-live birth groups (14.0% vs 17.2%, *P *= 0.010) and the clinical pregnancy and non-clinical pregnancy groups (14.5% vs 17.4%, *P *= 0.011).

## Discussion

Infertility is a major public health problem with a growing occurrence rate over the last decade. Despite rapid advancement in infertility treatments, there are still a large number of infertile women experiencing unsatisfying clinical outcomes from assisted conception treatment. For infertile couples seeking for ART treatment, the most important issue is to have a live birth; thus, it is mandatory to search for a method with maximum efficacy and minimum expense to improve the take-home baby rates. There were few published studies evaluating the impact factors, predictors or key factors of live-birth rate after ART, and a multivariate logistic regression analysis of factors affecting the live-birth rate of DOR patients in ART conceptions has not been previously performed. This retrospective cohort study addresses factors affecting the live-birth rate of DOR women undergoing fresh embryo transfer following IVF or ICSI, and provides valuable information for reproductive clinicians to improve the clinical ART management, and the following live-birth outcome.

It is widely accepted that age alone has an effect on fertility. Moreover, maternal age remains the most valuable factor predictive for the duration of reproductive life span [14]. For DOR women, our study demonstrated that the live-birth rate decreased with the increase of age, especially for those of ≥ 40 years old. We observed that there were statistically significant differences between the LB and NLB groups in both maternal and paternal age (*P *< 0.001, respectively). However, after adjusting for confounding variables, only maternal age remained different in the multivariate logistic regression model. Nowadays, socioeconomic changes have caused a growing trend for women all over the world to delay parenthood until later ages, which contributes to increased incidence of subfertility and an increasing number of advanced maternal age women opting for fertility treatments. Although ovary is the main determinant of female reproductive aging, uterine factors are also additional contributors [15]. Advanced maternal age is related to many adverse factors affecting the quality of oocyte, including cytoplasm (abnormalities of the cytoskeleton, reduction in mitochondrial number), nucleus (abnormal spindle formation, aneuploidy), and zona pellucida functions [16, 17]. In consistent of our study, Mutlu et al. reported in a prospective study including 192 infertile patients that age was independent predictor of live birth [18], which was closely associated with chromosomal abnormalities and mitochondrial DNA mutations in oocytes for women of advanced maternal age [19, 20]. For DOR women with infertility, the acceptable option is to seek assisted reproduction as soon as they are diagnosed, instead of long-term conception attempt without interfere or consultation from reproductive clinicians.

A case–control study reported that the basal T level presented a positive association with pregnancy outcome in women with DOR [21]. Conversely, there was no statistically significant difference in serum basal T level between the two study groups in our study (0.92 ± 1.12 vs 0.84 ± 1.00 nmol/L; *P *= 0.218). We also found that serum basal E_2_ levels were marginally higher in the NLB group in crude analysis (138.21 ± 90.02 vs 130.14 ± 97.57 pmol/L, *P *= 0.082), but the difference was not statistically significant. This finding is in accordance with a previous study which reported that serum E_2_ had no correlation with LB rate [22]. Baker et al. found a negative association between gonadotropin dose and LB after IVF treatment, which was consistent with our study [23]. Previous studies documented the influence of female BMI on IVF outcomes, reporting that BMI may affect pregnancy outcome independently [24, 25]. Of note, the present study showed that for DOR women, the LB group had statistically lower BMI (22.01 ± 3.11 vs 22.31 ± 2.87 kg/m^2^; *P *= 0.039). However, further regression analysis after adjusting for potential confounders showed that it was not an independent impact factor affecting the IVF-ET live-birth rate in women with DOR. Dokras reported a significantly higher risk for IVF cycle cancelation in obese patients, however, with no effect of BMI on clinical pregnancy or live-birth rate [26]. Disparities among different studies may be resorted to the differences in study designs, sample sizes, populations, physical conditions of women with DOR, and analytical methods.

We found that there were statistically significant differences in implantation rates in DOR women according to the outcomes of clinical pregnancy and live birth. However, the non-live birth group and the non-clinical pregnancy group had higher implantation rates compared to the live birth group (17.2% vs 14.0%, *P *= 0.010) and the clinical pregnancy group (17.4% vs 14.5%, *P *= 0.011), respectively. We speculate that the reason of higher implantation rates in the non-live birth group and the non-clinical pregnancy group could be explained by the proportion of age-specific miscarriages in older women, especially for those of ≥ 40 years old. Advanced maternal age women made up a large proportion in the non-live birth group (29.0% vs 10.5%, *P *< 0.001) compared to the live birth group, and the non-clinical pregnancy group (28.9% vs 15.9%, *P *< 0.001) compared to the clinical pregnancy group.

Recommendations have been generally intended for cleavage-stage embryos transferred on day 2 or 3 [27], while many reproductive medical centers prefer to transfer blastocyst-stage embryos [28]. To date, the optimal time for selecting embryo(s) to transfer is mixed. It was documented that the probability of live-birth was significantly higher after blastocyst-stage embryo transfer compared to cleavage-stage embryo transfer [29]. However, De Vos A et al. suggested that the choice of embryo transfer day seemed to play no roles on the chances of having a live-born child [30]. We observed that there was a significantly higher clinical pregnancy and live-birth rates among patients with embryos transferred on day 3 compared with day 2.

Furthermore, there were significantly higher clinical pregnancy and live-birth rates among patients transferred with three or two embryos compared to those with one embryo; however, the multiple pregnancies and miscarriages rates also increased simultaneously. Ideally, the aim of ART is to achieve a healthy singleton gestation [31, 32]. The American Society of Reproductive Medicine (ASRM)’s guidelines for the number of embryos to be transferred have been further refined in continuing efforts to decrease the number of multiple pregnancies [33], since multiple gestations lead to a higher risk of maternal and live-birth complications [34]. Among patients younger than 35 years of age undergoing IVF treatment with a favorable prognosis, a single embryo transfer has the highest chance of good perinatal outcome [35]. Therefore, based on our results, we do not recommend women with DOR to transfer high embryo numbers merely to improve clinical pregnancy rate, while regardless of live-birth rates or following obstetric outcomes.

Since the present study is a 5-year, single-center retrospective cohort study, there were limitations which cannot be ignored, in spite of a relatively large number of subjects reviewed. The potential unmeasured confounding factors, insufficient data concerning the definite diagnosis of DOR, and the retrospective design itself may all add to the uncertainty of the results. Furthermore, we only evaluated the impact factors affecting the live-birth rate of fresh embryo transfer cycles in DOR women and, hence, the results cannot be extrapolated to frozen embryo transfer cycles or normal ovarian reserve women. The study was conducted in a single reproductive medical center with standardized laboratory techniques, ovarian stimulation protocols, and embryo transfer procedures. Therefore, multi-center based randomized controlled trials (RCTs) with better planning are suggested in the future study. Further research with in vivo and in vitro models should be undertaken to explore the molecular mechanisms underlying this variation in live-birth outcome of infertile women with DOR in the future. Moreover, we will explore the intriguing association between blastocyst-stage embryos transfer and live-birth rates, as well as perinatal and neonatal outcomes in DOR women further in the follow-up study.

In summary, after adjusting for potential confounding factors, we observed that the maternal age, the embryo stage, and the number of embryos transferred were the independent impact factors affecting the IVF or ICSI live-birth rates in women with DOR. We do not recommend increasing the number of embryos transferred blindly to improve pregnancy rates. The results of this study are helpful for women with DOR and reproductive physicians to have a better understanding of the importance of female age, and the stage and number of embryos transferred on the specific ART outcomes. Efforts should be focused on factors that seem to be mostly predictive of live birth to improve take-home baby rates in the future.
